# Functional *COMT* Val158Met Polymorphism, Risk of Acute Coronary Events and Serum Homocysteine: The Kuopio Ischaemic Heart Disease Risk Factor Study

**DOI:** 10.1371/journal.pone.0000181

**Published:** 2007-01-31

**Authors:** Sari Voutilainen, Tomi-Pekka Tuomainen, Maarit Korhonen, Jaakko Mursu, Jyrki K. Virtanen, Pertti Happonen, Georg Alfthan, Iris Erlund, Kari E. North, M.J. Mosher, Jussi Kauhanen, Jari Tiihonen, George A. Kaplan, Jukka T. Salonen

**Affiliations:** 1 Research Institute of Public Health, University of Kuopio, Kuopio, Finland; 2 Atherosclerosis Research Unit, University of Kuopio, Kuopio, Finland; 3 Department of Public Health and Clinical Nutrition, University of Kuopio, Kuopio, Finland; 4 Biomarker Laboratory, National Public Health Institute, Helsinki, Finland; 5 Department of Epidemiology, School of Public Health, University of North Carolina, Chapel Hill, North Carolina, United States of America; 6 Department of Forensic Psychiatry, University of Kuopio, Kuopio, Finland; 7 Department of Epidemiology, School of Public Health, University of Michigan, Ann Arbor, Michigan, United States of America; 8 Oy Jurilab Ltd, Kuopio, Finland; University of Giessen, Germany

## Abstract

**Background:**

The role of circulating levels of total homocysteine tHcy in the development of coronary heart disease (CHD) is still under debate. One reason for conflicting results between previous studies on homocysteine and heart diseases could be consequence of different interactions between homocysteine and genes in different study populations. Many genetic factors play a role in folate-homocysteine metabolism, like functional polymorphism (Val108Met) in the Catechol-*O*-methyltransferase (*COMT)* gene.

**Methodology and Findings:**

Our aim was to examine the role of *COMT* Val158Met polymorphism and interaction of this polymorphism with serum tHcy and folate concentration on the risk of acute coronary and events in middle-aged men from eastern Finland. A population-based prospective cohort of 792 men aged 46–64 years was examined as part of the Kuopio Ischaemic Heart Disease Risk Factor Study. During an average follow-up of 9.3 years, there were 69 acute coronary events in men with no previous history of CHD. When comparing the* COMT* low activity genotype with the others, we found an age and examination year adjusted hazard rate ratio (HRR) of 1.73 (95% confidence interval (CI), 1.07–2.79), and an age, examination year, serum LDL and HDL cholesterol, and triglyceride concentration, systolic blood pressure and smoking adjusted HRR of 1.77 (95% CI, 1.05–2.77). Although serum tHcy concentration was not statistically significantly associated with acute coronary events (HRR for the highest third versus others 1.52, 95% CI, 0.93–2.49), subjects with both high serum tHcy and the *COMT* low activity genotype had an additionally increased adjusted risk of HRR 2.94 (95% CI 1.50–5.76) as compared with other men.

**Conclusions:**

This prospective cohort study suggests that the functional *COMT* Val158Met polymorphism is associated with increased risk of acute coronary events and it may interact with high serum tHcy levels.

## Introduction

Nutrition has a significant role in the prevention of many chronic diseases such as cardiovascular disease (CVD), cancers and degenerative brain diseases. Major risk and protective factors in the diet are now known, however, new candidates in both categories are still being revealed. Especially, the associations between nutrition and genes in disease risk have not been studied extensively.

During the last years, elevated plasma/serum total homocysteine (tHcy) has been one of the most intensely studied risk factors for CVD [1], [2]. We have shown in the Kuopio Ischaemic Heart Disease Risk Factor (KIHD) Study that a high serum concentration and dietary intake of folate are associated with a significantly lower risk of acute coronary events [3]–[5], while an elevated plasma tHcy concentration was not significantly associated with an increased risk [5]–[6].

There are some well-known genetic factors that have a role in the folate-Hcy metabolism, like methylenetetrahydrofolate reductase (MTHFR) and cystathionine ß-synthase (CBS) [7], [8]. There are also some less studied genetic factors in that metabolism, which could have a role in Hcy-CVD association. Catechol-*O*-methyltransferase (COMT) is an enzyme that catalyzes the *O*-methylation of various compounds, like catechol estrogens and dietary polyphenols, using S-adenosylmethionine (SAM) as the methyl donor and has also a role in dopamine inactivation. A common functional polymorphism (Val108Met) in the *COMT* gene is associated with a three- to four-fold variation in enzyme activity [9], [10]. The low activity genotype (Met158Met) has been associated with heavy alcohol use [11] and some other psychiatric disorders, such as bipolar disorder and schizophrenia [12].

A unifying hypothesis that hyperhomocysteinemia may exert its pathogenic effects largely through metabolic accumulation of SAH, a potent non-competitive inhibitor of COMT-mediated methylation metabolism of various catechol substrates was recently proposed [10]. Therefore we wanted to test the hypothesis that the functional polymorphism Val108Met in *COMT* gene could modify the coronary event risk, and that the effect is further strengthened by elevated tHcy.

## Materials and Methods

### Study design and population

The Kuopio Ischaemic Heart Disease Risk Factor (KIHD) Study is an ongoing prospective population-based cohort study designed to investigate risk factors for CVD, atherosclerosis and related outcomes in middle-aged men from eastern Finland [13], a population with one of the highest recorded rates of coronary heart disease (CHD). A total of 2682 participants (82.9% of those eligible), aged 42, 48, 54, or 60 years, were enrolled in the study between 1984 and 1989. The baseline examinations for the present prospective cohort study were carried out during 1991 to 1993. Out of a total of 1229 men eligible for the study, 52 had died, or suffered severe illness, or had migrated from the region, and 139 could not be contacted or refused to participate. Of the remaining 1038 men, data on serum folate and tHcy concentration and *COMT* genotype were available for 1012 men. As previous disease may affect circulating levels of tHcy and dietary intake of nutrients, men with prevalent CHD (n = 220) were excluded from the main analyses of this study. All participants signed a written informed consent. The Research Ethics Committee of the University of Kuopio has approved the KIHD study protocol.

### Measurements

The subjects came to give venous blood samples between 8 and 10 in the morning. They were instructed to abstain from ingesting alcohol for three days and from smoking and eating for 12 hours. After the subject had rested in the supine position for 30 minutes, venous blood samples were collected into vacuum tubes (Venoject; Terumo, Leuven, Belgium). No tourniquet was used. Blood for folate and cholesterol determination and for lipoprotein separation was drawn into serum tubes.

*COMT* polymorphism is generated by the presence of guanine or adenine at nucleotide 475 encoding a valine (Val) or methionine (Met) at codon 158 [14]. The polymorphism results in three different genotypes: homozygous for the low activity allele (Met/Met), homozygous for the high activity allele (Val/Val), and heterozygous (Met/Val). DNA was extracted from 10 ml of venous EDTA blood using standard salting-out or phenol-chloroform assays. *COMT* genotypes were determined by restriction fragment length polymorphism (RFLP) analysis from the DNA by an investigator unaware of the phenotype. A 179 base-pair polymerase chain reaction product was generated using the forward 5′-CTG CTG GAG CTG GGG GCC GAC-3′ and reverse 5′-AGG TCT TCA GGA ATG C-3′ primers. The polymerase chain reaction product was treated with HSP 92 II restriction endonuclease. The diagnostic bands were 179 (Val) and 139 (Met).

The serum tHcy concentration was analyzed by HPLC in 2001 at the National Public Health Institute, Helsinki, Finland [15]. The coefficients of variation (CV) between batches (n = 30) for two pooled serum samples were 4.3% and 5.4%.

Serum folate and vitamin B_12_ concentrations were measured by radioimmunoassay (Quantaphase II, Bio-Rad, Hercules, California, USA). These measurements were carried out in 1998 from serum samples collected during 1991–3 and kept frozen at −80°C. The between-batch CV of quality control serums (Lyphochek Immunoassay Plus Control levels 1, 2, 3, Bio Rad Laboratories, ECS Division, Anaheim, California, USA) for folate levels of 5.5, 13.4 and 23.6 nmol/L were 6.4, 6.7 and 6.7%, respectively (n = 16).

Serum lipoproteins were separated from fresh serum samples by combined ultracentrifugation and precipitation [16]. Serum total, LDL and HDL cholesterol (Kone Instruments, Espoo, Finland) and triglyceride (Boehringer Mannheim, Mannheim, Germany) concentrations were determined enzymatically with an autoanalyzer (Kone Specific, Kone Instruments, Finland).

Two trained nurses measured resting blood pressure with a random-zero mercury sphygmomanometer (Hawksley, United Kingdom). The measuring protocol included, after supine rest of five minutes, three measurements in the supine, one in the standing and two in the sitting position at five minute intervals. The mean of all six measurements was used as the systolic and diastolic blood pressure. Body mass index (BMI) was computed as the ratio of weight to the square of height. Assessment of smoking and ischemic disease in the family was carried out as described previously [17].

### Ascertainment of follow-up events

The province of Kuopio participated in the multinational MONICA (MONItoring of Trends and Determinants of Cardiovascular Disease) project [18], in which detailed diagnostic information of all heart attacks that occurred by December 1992 was collected prospectively. Diagnostic classification was made by the FINMONICA coronary registry group [18]. Data on acute coronary events between January 1993 and December 2002 were obtained by computer linkage to the national hospital discharge register and classified using identical diagnostic criteria, based on symptoms, cardiac enzymes and electrocardiographic findings as documented previously [18]. The average follow-up time was 9.3 years. If multiple non-fatal events occurred during the follow-up, the first event for each subject was considered as end point for the analyses. According to the diagnostic classification of the events there were 43 definite and 17 possible acute myocardial infarctions (AMI) and 9 typical prolonged chest pain episodes in men free of prior CHD at study baseline, a total of 69 events without history.

### Statistics

The data are expressed as means±SD. Means were compared by analysis of variance (ANOVA). The subjects were classified into thirds according to their serum folate and tHcy concentration and those in the highest third were compared with those in the two lower thirds. The relationship of serum folate and tHcy with the risk of acute coronary events in *COMT* genotypes was analyzed using the Cox proportional hazards models. We used age, examination year, serum HDL and LDL cholesterol and triglycerides, mean systolic blood pressure and smoking as covariates in the main analysis.

Interactions between genotype and serum tHcy and genotype and folate on the risk of acute coronary events were tested on both the multiplicative and additive scales. First, the ]multiplicative interaction between genotype and serum tHcy was assessed by the use of a product term that was serum tHcy ≥11.3 µmol/L * *COMT* low activity (Met158Met) genotype. Where the estimated β coefficient for the product term does not equal zero or the respective hazard rate ratio (HRR) does not equal one, a multiplicative interaction exists. An additive interaction was assessed using the estimated relative excess risk for interaction (RERI) [19]. Briefly, RERI = HRR_11_−HRR_10_−HRR_01_+1, where HRR_11_ refers to the hazard rate ratio of acute coronary event associated with both genotype and serum tHcy, HRR_10 _and HRR_01_ are hazard rate ratios of acute coronary event associated with genotype or serum tHcy. The estimated RERI value that does not equal zero suggests an additive interaction. The interactions between genotype and folate were assessed in the same way. The product term for serum folate was serum folate ≤8.3 nmol/L * COMT Met158Met genotype.

The confidence intervals (CI) were estimated on the basis of the assumption of asymptotic normality of the estimates. All tests of significance were two-sided. P-values <0.05 were considered significant. Data were analyzed using SPSS 11.0 for Windows (SPSS Inc., Chicago, IL, USA).

## Results

### Characteristics

At the beginning of follow-up, the mean age of the study men was 55.4 years. During an average follow-up time of 9.3 years, 69 men with no previous CHD at baseline suffered an acute coronary event. Those subjects who suffered an acute coronary event had higher systolic blood pressure, serum total and LDL cholesterol and triglycerides and a higher prevalence of ischemic heart disease in the family, and also had lower serum folate concentration than the other subjects (Table 1).

**Table 1:** Baseline characteristics of the study subjects*

Characteristic	All subjects (n = 792)	Subjects who developed an acute coronary event (n = 69)	Other subjects (n = 724)	*p*-value for difference in means
Serum tHcy, µmol/L	10.8±3.4	11.0±3.4	10.8±3.3	0.622
Serum folate, nmol/L	10.35±3.93	9.34±3.93	10.45±3.92	0.025
Mean age, y	55.4±6.6	56.3±6.6	55.3±6.6	0.197
BMI, kg/m^2^	27.5±3.6	28.2±3.3	27.4±3.6	0.101
Systolic blood pressure, mmHg	135±17	141±15	135±17	0.003
Total cholesterol, mmol/L	5.50±0.90	5.79±0.83	5.47±0.90	0.005
LDL cholesterol, mmol/L	3.91±0.81	4.14±0.72	3.89±0.81	0.012
HDL cholesterol, mmol/L	1.11±0.29	1.08±0.30	1.12±0.28	0.285
Triglycerides, mmol/L	1.57±1.03	1.89±1.42	1.54±0.98	0.007
Current Smokers, %	26.8	31.8	26.3	0.280
IHD in family, %	50.3	66.7	48.7	0.004

Abbreviations: BMI = body mass index; HDL = high density lipoprotein; IHD = ischemic heart disease; LDL = low density lipoprotein; tHcy = total homocysteine concentration.

* Data are expressed as the mean±SD unless otherwise indicated.

The distribution of the *COMT* genotypes was consistent with the Hardy-Weinberg equilibrium. The frequency of the Val/Val, Val/Met and Met/Met genotypes in healthy controls was 22.0, 50.3 and 27.7%, respectively (*p* = 0.78). In the whole study population the prevalence of heterozygosity and homozygosity of *COMT* Met158 were 49.4% and 28.9%, respectively. Table 2 exhibits baseline characteristics of the study subjects according to the *COMT* genotypes. There was no significant difference in by age, serum HDL, LDL or triglycerides, systolic blood pressure or smoking status in different *COMT* genotypes. There was also no statistically significant association between* COMT* genotypes with either serum tHcy or folate levels.

**Table 2:** Characteristics of the study subjects according to the catechol-o-methyltransferase (*COMT*) genotypes*

Characteristic	*COMT* ^Val158Val^ (n = 172)	*COMT* ^Val158Met^ (n = 391)	*COMT* ^Met158Met^ (n = 229)	*p-v*alue
Serum tHcy, µmol/L	11.0±4.4	10.7±3.2	10.9±2.6	0.521
Serum folate, nmol/L	10.3±4.2	10.4±3.8	10.3±4.0	0.927
Age, y	55.4±6.6	54.9±6.4	56.1±7.0	0.086
BMI, kg/m^2^	27.4±3.5	27.3±3.5	27.9±3.7	0.147
Systolic blood pressure, mmHg	134±15	135±17	136±17	0.397
Total cholesterol, mmol/L	5.63±0.90	5.43±0.93	5.52±0.84	0.056
LDL cholesterol, mmol/L	4.02±0.80	3.85±0.83	3.93±0.76	0.060
HDL cholesterol, mmol/L	1.12±0.29	1.09±0.26	1.14±0.33	0.061
Triglycerides, mmol/L	1.55±0.87	1.57±0.98	1.58±1.23	0.944
Current smokers, %	26.9	26.6	27.1	0.991
IHD in family, %	53.5	47.3	52.8	0.262
Subjects who developed an acute coronary event, %	8.0	7.0	13.0	0.041

Abbreviations: BMI = body mass index; HDL = high density lipoprotein; IHD = ischemic heart disease; LDL = low density lipoprotein; tHcy = total homocysteine concentration.

* Data are expressed as the mean±SD unless otherwise indicated.

### Serum homocysteine and folate and the risk of acute coronary event

Serum tHcy concentration was not significantly associated with the risk of acute coronary event. Men with high serum tHcy concentrations (highest third, ≥11.3 µmol/L) had a 1.41-fold (95% CI, 0.87–2.29, *p* = 0.164) risk of acute coronary events after adjusting for age and examination year and 1.52-fold (95% CI, 0.93–2.49, *p* = 0.092) risk after adjusting for age, examination year, serum HDL and LDL cholesterol and triglyceride concentration, systolic blood pressure and smoking as compared with men with lower serum tHcy. Additional adjustment with serum folate decreased this risk to 1.32 (95% CI, 0.80–2.18, *p* = 0.283). Subdividing serum tHcy into quarters or adjustment for other dietary or lifestyle factors did not reveal significant associations between tHcy and coronary events.

A low serum folate concentration (lowest third, ≤8.3 nmol/L) was associated with a significantly higher risk of acute coronary events. In the Cox model adjusted for age and examination years, and serum LDL- and HDL- cholesterol and triglycerides, systolic blood pressure and smoking, the HRR for those in the lowest serum folate third was 1.72 (95% CI 1.06–2.79, *p* = 0.028) as compared with others. Adjustment for other nutritional or lifestyle factors did not substantially attenuate this association.

### *COMT* low activity Met158Met genotype and the risk of acute coronary event

The *COMT* low activity genotype was associated with a higher risk of acute coronary events. In the comparison of the men with the *COMT* Met158Met genotype with the others, we found age and examination year adjusted HRR of 1.73 (1.07–2.79, *p* = 0.025), and age, examination year, serum HDL and LDL cholesterol, and triglyceride concentration, systolic blood pressure and smoking adjusted HRR of 1.77 (1.05–2.77, *p* = 0.030).

### Interactions between serum homocysteine and folate, *COMT* Met158Met genotype and acute coronary event risk

Table 3 presents the adjusted HRR's and 95% CI's of incident coronary events associated with gene-tHcy and gene-folate interactions. HRR's for coronary events were 1 for individuals with ‘non-risk’ genotype: subjects with *COMT* Val/Met genotype and either low serum tHcy (lowest third) for homocysteine-gene analyses or high serum folate (highest third) for folate – gene interaction analyses. HRR for interaction and RERI with 95% CI's are also displayed.

**Table 3:** Adjusted HRR and 95% CI of Incident Acute Coronary Events Associated with the *COMT* Genotype*, Plasma tHcy and Serum Folate and Gene-Homocysteine and Gene-Folate Interactions

Homocysteine, exposure definition									
	High tHcy, *COMT* Val158Met or Val158Val		Low tHcy with *COMT* Met158Met		High tHcy with *COMT M*et158Met		Multiplicative interaction		Additive interaction
	HRR (95% CI)	*p-value*	HRR (95% CI)	*p-value*	HRR (95% CI)	*p-value*	HRR_i_ (95% CI)	*p-value*	RERI (95% CI)
Model 1†	1.15 (0.60–2.20)	0.68	1.42 (0.75–2.68)	0.28	2.60 (1.35–5.03)	0.004	1.60 (0.60–4.25)	0.35	1.03 (−0.30–2.36)
Model 2‡	1.20 (0.62–2.30)	0.59	1.34 (0.70–2.56)	0.37	2.94 (1.50–5.76)	0.002	1.84 (0.68–4.95)	0.23	1.40 (−0.43–3.23)
**Folate, exposure definition**									
	**Low fol with** * COMT* ** Val158Met or Val158Val**		**High fol with ** ***COMT*** ** Met158Met**		**Low fol with ** ***COMT M*** **et158Met**		**Multiplicative interaction**		**Additive interaction**
	**HRR (95% CI)**	***p-value***	**HRR (95% CI)**	***p-value***	**HRR (95% CI)**	***p-value***	**HRR_i_ (95% CI)**	***p-value***	**RERI (95% CI)**
Model 1†	1.95 (1.05–3.63)	0.036	2.11 (1.11–4.01)	0.022	2.67 (1.28–5.59)	0.009	0.65 (0.25–1.73)	0.39	−0.39 (−2.60–1.83)
Model 2‡	2.05 (1.10–3.85)	0.025	2.07 (1.09–3.94)	0.027	2.80 (1.33–5.90)	0.007	0.66 (0.25–1.76)	0.41	−0.32 (−2.82–2.18)

Abbreviations: CI, confidence interval; COMT, catehol-*O*-methyltransferase; fol, serum folate concentration; HRR, hazard rate ratio; HRR_i_, hazard rate ratio for a multiplicative interaction; RERI, relative excess risk for interaction; tHcy, serum total homocysteine concentration.

* *COMT* low activity Met158Met is ‘at-risk’ genotype for this study. The comparison group for the HRRs in the first three columns of the table is the same, i.e. subjects with lower plasma tHcy (<11.3 µmol/L) with *COMT* Met158Val or Val158Val genotype.

† Model 1 is adjusted for age and examination years.

‡ Model 2 is adjusted for age, examination years, serum HDL and LDL cholesterol and triglycerides, systolic blood pressure and smoking

**Figure 1:**
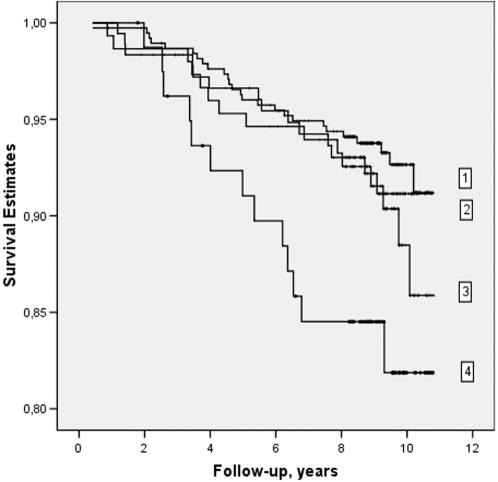
Kaplan-Meier survival curves for 1) study subjects with lower serum total homocysteine (tHcy<11.28 µmol/L) and no *COMT* Met158Met genotype, 2) study subjects with higher serum tHcy (≥11.28 µmol/L) and no *COMT* Met158Met genotype, 3) study subjects with lower serum tHcy and *COMT* Met158Met genotype, and 4) study subjects with higher serum tHcy and *COMT* Met158Met genotype.

When comparing men with both high plasma tHcy (highest third) and *COMT* Met158Met genotype with the men with lower serum tHcy and *COMT* Met158Val or Val158Val genotype, we found age and examination year adjusted HRR of 2.60 of acute coronary event (95% CI 1.35–5.03, *p* = 0.004) (Table 3). After adjusting for age, examination years, serum HDL and LDL cholesterol and triglycerides, systolic blood pressure and smoking the HRR was 2.94 (95% CI 1.50–5.76, *p* = 0.002). There was no significant association between high serum tHcy and acute coronary events in men with other *COMT* genotypes. In Table 3, however, the results suggest no statistically significant multiplicative nor additive interaction between *COMT* genotype and serum tHcy (*p*-values >0.05). Figure 1 presents Kaplan-Meier survival curves for 1) study subjects with lower serum tHcy (<11.28 µmol/L) and no *COMT* Met158Met genotype, 2) study subjects with higher serum tHcy (≥11.28 µmol/L) and no *COMT* Met158Met genotype, 3) study subjects with low serum tHcy and *COMT* Met158Met genotype, and 4) for the study subjects with higher serum tHcy and *COMT* Met158Met high-risk genotype.

In order to study the interaction between serum folate levels and *COMT* genotypes, we conducted similar analyses with serum folate. Men with both low serum folate and *COMT* Met158Met genotype had a adjusted HRR of 2.80 (95% CI 1.33–3.90, *p* = 0.007) when compared with men having high serum folate and other *COMT* genotypes (Table 3). Nevertheless, the interaction between *COMT* genotype and serum folate was not statistically significant either on the multiplicative or additive scale (*p*-values >0.05).

## Discussion

The main finding of this study is that the common functional Met158Met polymorphism of the *COMT* gene is an independent risk factor for acute coronary events in Finnish men and it may also interact with serum tHcy to increase the risk of coronary events further. Recently Eriksson and colleagues we have shown opposite results. In their prospectively followed hypertensive cohort of 174 patients and 348 controls the low activity genotype reduces the risk of myocardial infarction [20]. They also noticed that men who were homozygous for the low activity allele of COMT had increased serum levels of estradiol, and concluded that the altered estrogen status could be involved in this effect. In that study 74% of study subjects were men and interaction between COMT genotype and homocysteine were not studied.

It is biologically plausible that the *COMT* variant directly modifies the association between high serum tHcy levels and risk of CHD because of prior evidence suggests that COMT is one of the key enzymes in the methionine-homocysteine metabolism [10]. An alternative explanation for our findings is that other genes on chromosome 22 with functional mutations are in linkage disequilibrium with the *COMT* variant evaluated herein. Homocysteine is a sulphur-containing amino acid which is converted to SAM, formed only from the dietary essential amino acid methionine. Via the action of SAM adenosyltransferase, methionine is converted to SAM, which is the major biological methyl donor required for numerous cellular processes, including the formation of creatine and methylation of phospholipids. These reactions are catalyzed by various methyltransferases, such as COMT, which demethylates s-adenosylmethionine to SAH, the immediate precursor of homocysteine. Recently a unifying hypothesis that hyperhomocysteinemia may exert its pathogenic effects largely through the metabolic accumulation of SAH, which is a potent non-competitive inhibitor of COMT mediated methylation of various catechol substrates, such as catecholamines and catechol estrogens has been proposed [10]. In the case of endogenous catecholamines in peripheral tissue, inhibition of their methylation by SAH could theoretically result in elevation of blood or tissue levels of catecholamines, and consequently, over-stimulation of the cardiovascular system. The endogenous catecholamines are also potentially reactive molecules. Our findings from the KIHD Study are in line with recent hypothesis; it seems that a moderate increase of serum tHcy (which may cause a moderate inhibition of the COMT activity) may not reduce the level of COMT activity below the minimally-required level in the whole study population. However, in people already with a compromised COMT activity, the pathogenic consequences would be more readily detectable, and risk of acute coronary events could increase.

The mean serum tHcy concentrations are rather similar to other studies from Finland [21], [22], a country where severe or moderate hyperhomocysteinemia is relatively uncommon. The negative tHcy-AMI findings in another Finnish study were proposed to be due to the low prevalence of mutations predisposing to hyperhomocysteinemia in the Finnish population [22]. Indeed, homocystinuria is a hereditary disorder which is extremely rare in Finland [23].

Our study is limited by the small number of outcome events, reducing the power and preventing testing for interaction analyses or including multiple covariates in the models. The major strength of this study is its prospective population-based study design with a long follow-up period.

In summary, although a high circulating level of tHcy does not appear to predict acute coronary events in healthy men living in eastern Finland, high tHcy could be a modest risk factor in subpopulations with predisposing gene mutations, such as the *COMT* low activity Met158Met genotype. Additional studies will be required to provide more information about the *COMT* gene in cardiovascular health.
